# Application and comparative analysis of Intelligent Monitoring Technology for Grouted Pile Construction based on abaqus

**DOI:** 10.1038/s41598-024-59653-9

**Published:** 2024-04-22

**Authors:** Rong Wu, Yangyang Jiang, Shunzhi Zhao, Min Chen, Shiqiang Shang, Xuedong Lang

**Affiliations:** Yunnan Airport Construction and Development Co., LTD, Kunming, 650000 China

**Keywords:** Bored pile, Intelligent monitoring, Combined positioning technology, Abaqus comparative analysis, Perpendicularity, Energy science and technology, Engineering

## Abstract

In order to realize the intelligent monitoring of the high-precision positioning of the hole position and the real-time control of the verticality of the pile, an intelligent monitoring system was developed based on the combination positioning technology of BDS and UWB and the biaxial tilt sensor, and the numerical simulation and comparative analysis of the verticality of the pile were carried out by abaqus. The deviation of pile foundation in different directions and the deviation of pile body are controlled by the monitoring system, and abnormal warning is made when the deviation exceeds the permissible range.Through the application of intelligent monitoring system in the pile foundation engineering area of Changshui Airport, it is found that the plane offset and perpendicularity of all piles meet the standard requirements and the construction error is controlled at a small value. The results show that the application of intelligent inspection system can not only ensure the construction quality of pile foundation, but also meet and improve the level of digitization and information technology of smart construction site.

## Introduction

With the rise of the central part of the country, the continuous promotion of the revitalization strategy of the northeast and the proposal of the "Belt and Road" initiative, the speed and quantity of infrastructure construction are still on a large scale. As an important part of engineering construction, foundation engineering plays a decisive role in engineering construction quality control. Pile foundation is widely used in all kinds of foundation engineering due to its advantages of high strength, high bearing capacity and low deformation^1–3^. Hammer-hit pile, static pile pressing, vibratory pile sinking and bored pile are the four most commonly used pile foundations at present. Compared with other pile forming methods, bored pile has the characteristics of strong single pile bearing capacity, low pollution, low noise and no influence of groundwater level, etc., and is widely used in semi-saturated soil and complex environmental geology^4,5^. However, the construction quality of bored piles cannot be monitored in real time and accurately while they are used in large quantities. Because the pile foundation belongs to underground hidden engineering, the pile quality of bored pile can not be controlled at the same time of construction, even if the pile foundation problem is found after sampling inspection, it is difficult to remedy. Due to the shortcomings of the traditional monitoring methods used at present, they can not meet the needs of the existing construction projects.

In order to solve the problem that the construction process of cast-in-situ pile cannot be monitored in real time, relevant scholars put forward different construction schemes from various angles. For example, Wu et al.^6^ built a smart site management platform through the combination of Beidou positioning technology, contact instrument assembly method, resonant tuning fork sensing technology, BIM technology, etc., which can effectively realize the intelligent management and control of grouting piles. Sun et al.^7^ tested the quality of bored pile through GZ-2S monitoring system. Based on the test data, they put forward suggestions on the optimization of relevant construction technology, thus improving the quality of bored pile. He et al.^8^ proposed a pile stress and deformation monitoring technology based on the distributed fiber optic model, which can accurately reflect the structure, deformation and stress characteristics of harbor pool cast-in piles, and evaluate the role of piles accordingly. Gao et al.^9^ used OFDR (optical frequency domain reflection fiber sensing) technology to conduct global monitoring of the deformation of the pile body, and analyzed the monitoring data and found that there was time lag effect in axial unloading of the pile. Zhang et al.^10^, based on the characteristics of different resistances of different media in cast-in piles, characterized the pile perfusion height through the fitting curve between the resistance and the probe, and built an intelligent system capable of monitoring the pile perfusion height.

The existing research has carried out the analysis and application of the construction intelligence of cast-in-place piles through different technologies and equipment. Intelligent construction includes automation, intelligence and information technology, which includes sensing technology to detect and feedback various parameters of the pile body itself during the construction process, such as the detection of pile strength and verticality. Secondly, the control technology is the core of intelligent construction, which deals with the construction parameters and status in real time to ensure the progress of the construction. The automation technology is an important means to realize the intelligent construction and the implementation of the pile construction automation greatly saves consumption. Information technology is used for information collection and transmission, such as the transmission of sensor information data to the computer to improve the efficiency of the pile construction. The development and application of the intelligent system for the high-precision real-time monitoring of the positioning of holes and the real-time control of pile verticality or offset still need to be further explored. In order to meet the requirements of intelligent monitoring of the construction of injected pile in the construction of engineering projects, this paper uses the combination positioning technology of BDS and UWB to develop a digital construction integrated management platform for the monitoring of the hole position and pile vertical, and realizes the visualization and early warning auxiliary functions of the construction process of injected pile. In order to realize the improvement of the level of digitization and information technology of engineering construction. At the same time, the application of intelligent monitoring system not only provides a reference for the construction of smart site, but also echoes the national initiative on the construction of digital China.

## Intelligent monitoring of bored pile construction

### Traditional monitoring methods

China's Ministry of Housing and Urban–Rural Development has made clear regulations on the construction procedures and processes of different types of pile foundations in foundation engineering, including pre-construction preparation, mid-term operation, process monitoring, post-inspection and safety management in the whole process^11^. Generally, the construction of bored pile foundation includes pay-off location, protection casing buried, drill drilling, pile hole setting, final hole monitoring, mud hole clearing, reinforcement cage laying, concrete pouring, etc.^12^. The monitoring of hole position and verticality of cast-in piles during construction is particularly important for pile foundation placement and pile body deviation, which not only affects the installation and lowering of steel cage in the later stage, but also reduces the bearing capacity of pile foundation. At the same time, a certain space should be arranged according to the external conditions to avoid the encroachment of pile main structure caused by inclined holes, which has certain requirements for the verticality of cast-in piles. In summary, it can be seen that in the construction process of bored pile, it is necessary to accurately grasp the plane position of pile and the deviation of pile body (verticality) during the hole-forming stage of pile foundation. Due to the many construction procedures of bored pile and the difficulty of on-site supervision, the existing monitoring methods have some shortcomings, such as the impact of aperture size and mud environment. The monitoring cannot be carried out at the same time as the hole forming construction and the operation method is complicated^13,14^. As shown in Table 1, it is part of the traditional pile foundation verticality monitoring method.

**Table 1:** Traditional monitoring methods of pile foundation verticality.

Monitoring methods	Apex angle measurement	Center fitting method	Ultrasonic method
Instrument and equipment	Inclinometer, centralizer	Umbrella aperture meter	Ultrasonic hole forming instrument
Scope of application	Pile diameter ≤ 3.0 m	Pile diameter ≤ 3.0 m	Pile diameter ≤ 0.6 m
Operability	Proceed in stages	After forming holes	Probe lifting operation

### Combining positioning technologies

The traditional monitoring method can not meet the high precision demand in the construction process of cast-in-situ pile. There are many kinds and quantities of engineering data to be monitored or construction parameters to be controlled in the process of borehole and pile formation. The existing monitoring methods have some problems such as low efficiency, manual error and inability to track in real time. The conventional drilling position of cast-in-place piles is determined by manual positioning or machine-assisted positioning, which makes the drilling rig easily deviate from the designed pile position due to lack of real-time monitoring data. Based on BDS (Beidou navigation and positioning System), some foundation projects monitored the whole process of pile foundation hole-formation positioning and laying^15–17^, and adopted Real-time kinematic (carrier phase differential positioning) technology in BDS to position the horizontal and elevation position of the target object, and converted the position of pile point from the coordinate position. To realize the automatic navigation and positioning of pile location, the positioning accuracy can meet the engineering requirements.

**Figure 1:**
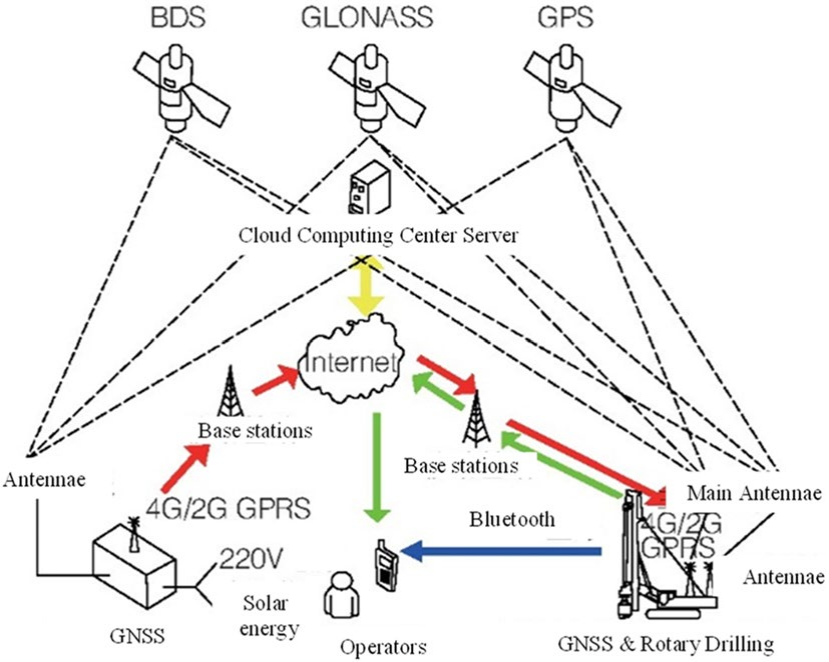
Schematic diagram of pile fixing by navigation and positioning system.

By installing the Beidou positioning directional antenna on the pile machine, combining with the network difference technology to achieve centimeter-level positioning, and combining with the strapdown inertial navigation algorithm, the positioning directional antenna installation position is calculated to the center of the drill, so as to realize the real-time calculation of the center position of the drill in the subsequent construction process and real-time comparison between the position and the design coordinates of the pile point. Then, the deviation between the current drill center and the design position of the pile point is obtained, and visualized on the vehicle terminal. Its working principle is shown in Fig. 1. The application of BDS technology can solve the problems such as the deviation of artificial placement point, the damage of point mark and the difficulty of finding point at night in the traditional construction process. Through a large number of engineering practices, it is found that although the application of BDS can greatly improve the efficiency of bored pile location, when using this technology, the satellite needs to receive target information and respond before positioning, and too much target information may cause signal blocking, thus affecting the hole location and layout of pile foundation.

**Figure 2:**
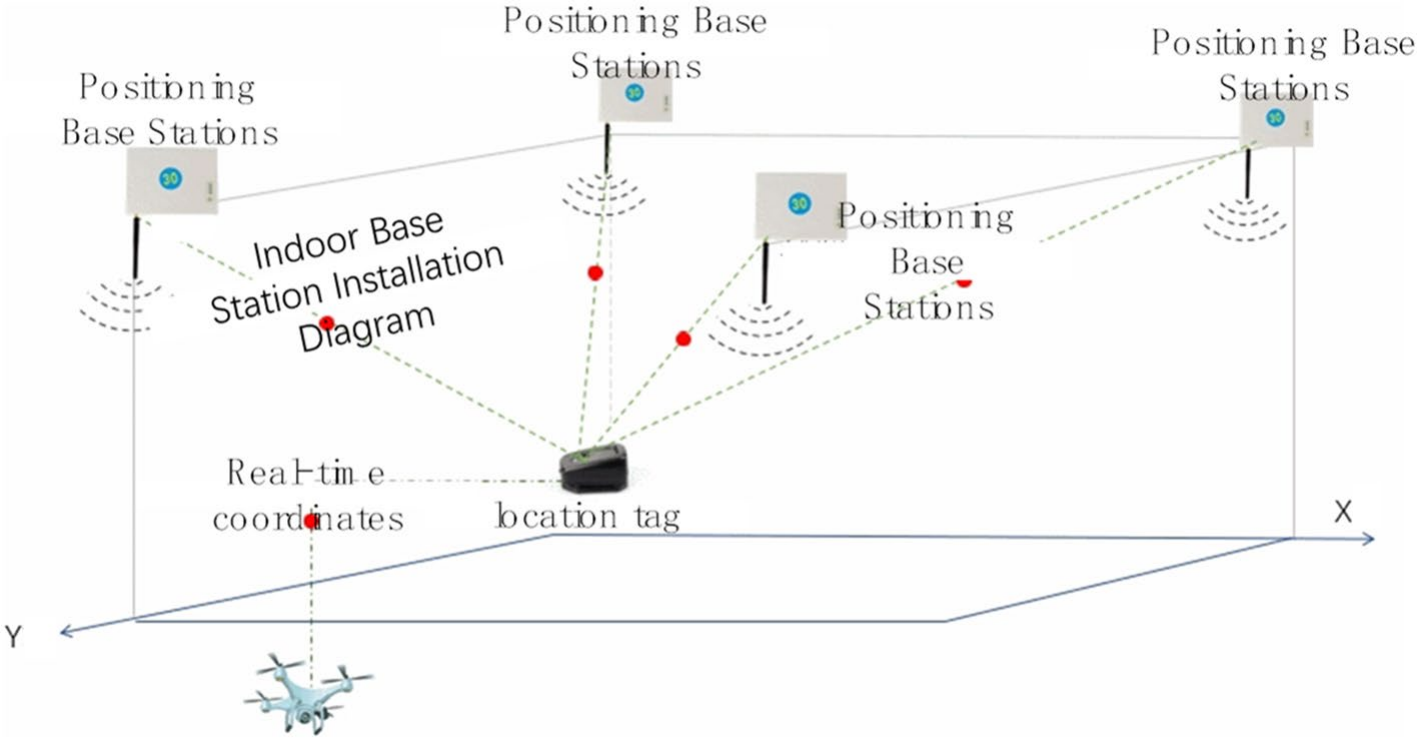
Schematic diagram of UWB technology.

In addition to the application of BDS, research is also being carried out to determine the position of pile points by Ultra Wideband (UWB) technology. UWB is a carrier-less communication technology, which uses non-sinusoidal narrow pulses in the nanosecond to microsecond level to transmit data, and transmits extremely low power signals over a wide spectrum. With the advantages of high transmission rate, strong security, low power consumption and high positioning accuracy, it is widely used in indoor positioning, and its positioning working mechanism is shown in Fig. 2. At present, UWB positioning technology has been applied in the smart construction site, through the deployment of a certain number of positioning base stations on the construction site, for personnel or equipment to wear a hard hat label card in the form of obtaining target location information. The positioning data from the UWB are combined with data from other sensors, and the relevant algorithms are utilized to assign weights and combine multiple positioning data to obtain more accurate positioning results. By combining these technologies and optimizing the way they work together, the device is able to provide more accurate and stable pile positioning work.

The deployment structure of the UWB system needs to be installed at three levels, namely, the software (server) level, the monitoring level, the positioning level and the acceptance level. The single positioning level of the UWB system is to lay positioning base stations and positioning labels, transmit information through the short-time pulse radio generated by the pulse generator, and install positioning labels on the pile foundation. The label communicates regularly with the base station in the range to complete the positioning ^18–20^.

In order to solve the shortcomings of using BDS or UWB technology alone, a combination positioning technology based on BDS and UWB can be built. Both BDS and UWB technologies are taken into account in the combination positioning technology, and RTK and time difference positioning technology in the core of the two technologies can complement each other well. In the combined positioning technology, the positioning of UWB system is replaced by BDS system, which makes the combined positioning technology significantly improve in many aspects compared with UWB technology: (1) The positioning information of pile foundation is transformed from timing communication to real-time communication; (2) The location range of pile foundation is changed from the range of base station to the whole construction area; (3) The positioning accuracy was increased from 30 to 2 cm.

**Figure 3:**
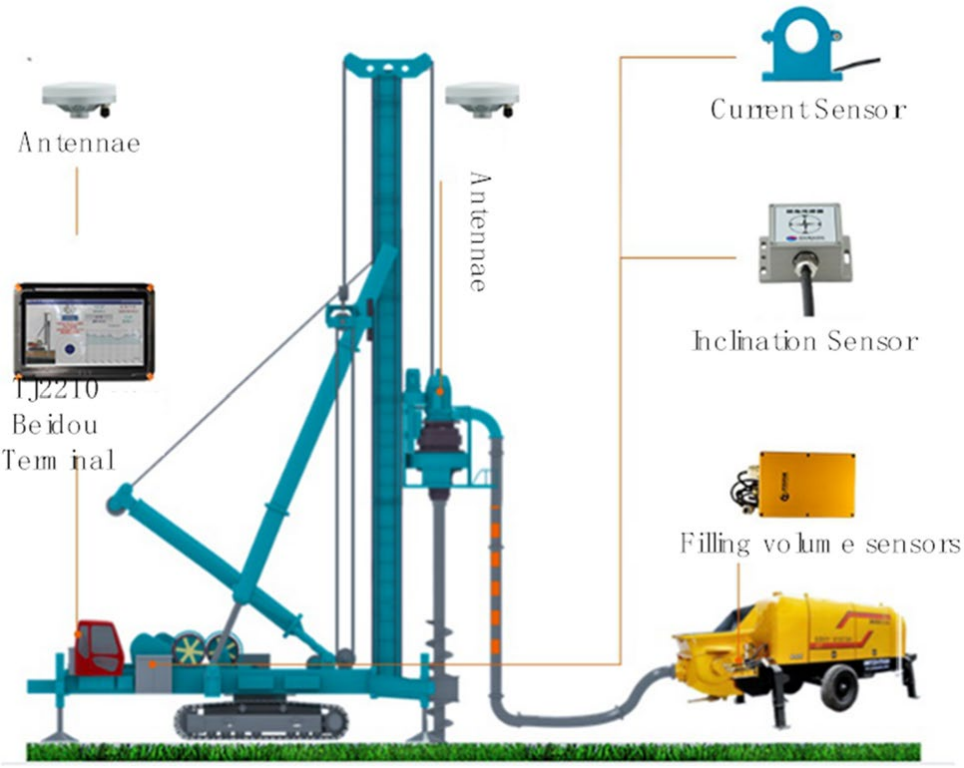
Schematic diagram of pile positioning by combination positioning technology.

The application of the combined positioning technology can strengthen the positioning stability of pile foundation, achieve the centimeter-level positioning accuracy of the drill rig, automatically guide the drill pipe to position, assist manual pile lofting, real-time monitoring of the drill bit moving position and the rotating direction of the pile machine, and improve the measurement and positioning accuracy. The combined working diagram is shown in Fig. 3. According to the construction characteristics of drill equipment, other high-precision sensor equipment can be deployed for the construction quality control of bored piles, such as the tilt sensor to monitor the verticality of pile foundation, and the post-detection can be transformed into in-process monitoring. Through monitoring data, real-time observation of construction status, adjustment of drilling equipment, pile tilt, etc., and then improve the quality of pile foundation.

### Intelligent monitoring system

According to the construction requirements of bored piles, a digital construction comprehensive management platform is developed to collect and analyze multi-source data to realize monitoring data query, engineering volume statistics, and construction quality management. At the same time, it has real-time alarm function for abnormal conditions of pile foundation. On-site managers can monitor and understand the construction status of single pile foundation in real time and obtain quality records of the whole process of single pile hole formation. The intelligent monitoring system of cast-pile construction is a part of the digital construction integrated management platform, and the intelligent monitoring system can simultaneously monitor the plane position of the pile and the verticality of the pile. The former realizes real-time monitoring of displacement data by combining positioning technology, and the latter realizes real-time monitoring of tilt data by installing inclination sensors. The construction information is collected by combining positioning technology and inclination sensor terminal and transmitted to the digital construction integrated management platform center for analysis, and real-time monitoring is carried out through the management platform.

**Figure 4:**
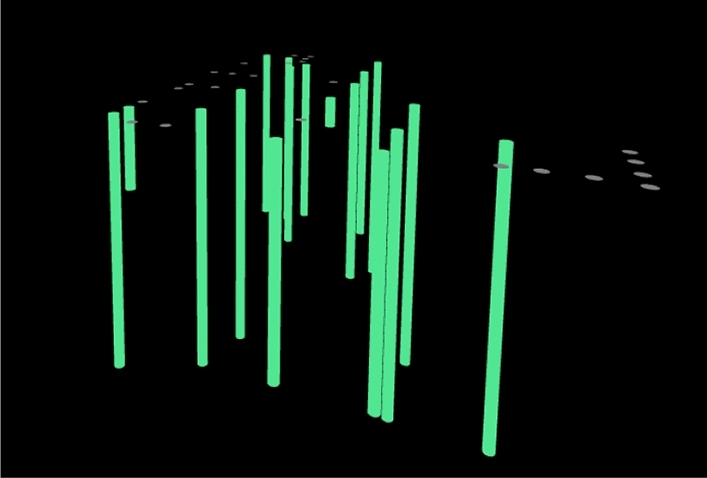
Dynamic three-dimensional view of pile points.

The intelligent monitoring platform for bored pile consists of four operation modules: basic information, early warning configuration, achievement display and early warning query, among which the achievement display and early warning query module are the core parts of the monitoring platform. The former is responsible for monitoring the whole process of the construction of cast-in-place piles, as shown in Fig. 4. The latter is responsible for the alarm work when the deviation exceeds the specification occurs in the construction process.

## Engineering application

### Project overview Yunnan province

**Figure 5:**
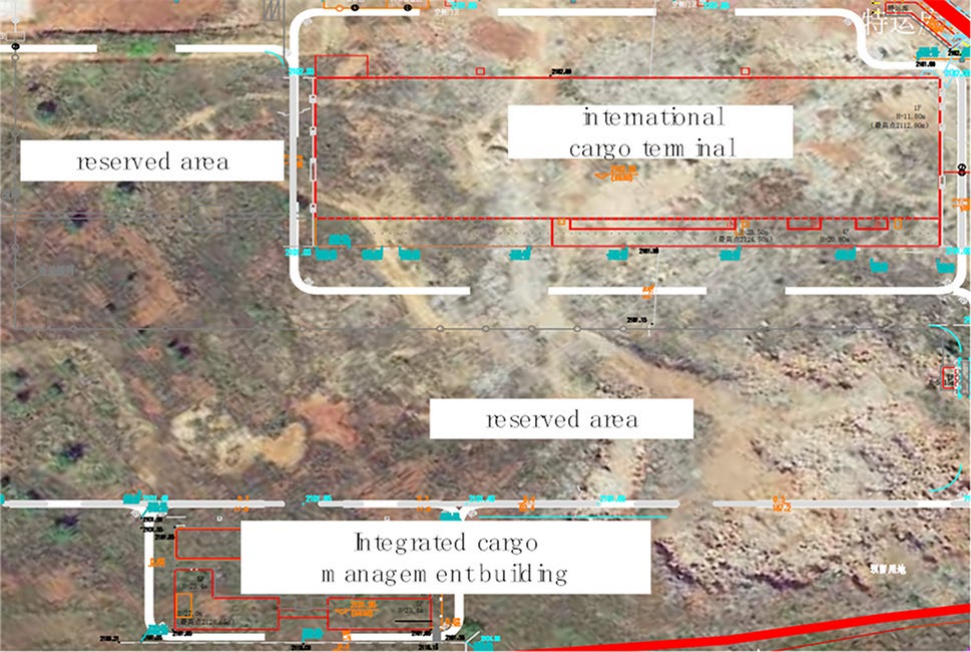
Aerial photo of T2 Terminal renovation and expansion of Kunming Changshui Airport.

Kunming Changshui International Airport T2 Terminal renovation and expansion of earth and rock foundation treatment and ancillary works-East Cargo area project EPC project general contract project construction site is located in the airport master plan of the east cargo area of the current construction land (central south area), the total construction area of the project 99,198.41 m^2^. The main facilities include domestic freight station, international freight station, special warehouse, fumigation room, bayonet, maintenance workshop, guard room, freight integrated management room (including shift dormitory, canteen) and outdoor engineering of freight area within the scope of the land (see Fig. 5). The pile foundation project is divided into two construction areas, namely the international freight station (20 pieces) and the freight integrated management room (202 pieces). There are 222 bored piles in the construction area of the project, and the number of pile foundations completed through the intelligent monitoring system has accounted for more than 10%. In order to analyze the role of the intelligent monitoring system in the pile laying process, two of the piles were selected in the international freight station (No. J1 and J2) and the freight integrated management room (No. H1 and H2), and the changes in the plane position and perpendicularity of the pile were analyzed. The traditional location monitoring methods used include the Global Positioning System (GPS). To a certain extent, they can meet the construction requirements, but they have some limitations in terms of accuracy requirements. For example, although GPS positioning is widely used under certain special circumstances, such as when encountering poor weather, the positioning accuracy may be affected. In addition, there are limitations in terms of real-time and data processing speed.

### Application analysis

**Figure 6:**
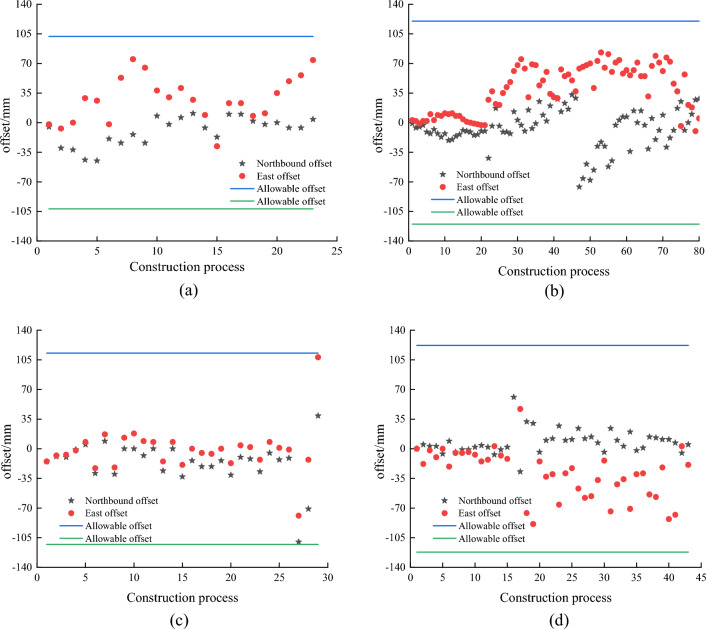
Construction process monitoring of cast-in-place pile.

The pile foundation digital system equipment is installed on the pile machine, wherein the positioning and orientation device is installed on both sides of the pile machine mast, and the biaxial inclination sensor is installed on the mast. The whole construction process of four cast-in-place piles in two pile areas was monitored by the intelligent monitoring system, and the changes in the plane positions of pile foundation holes were analyzed based on the monitoring data obtained by the combined positioning technology. The results are shown in Fig. 6.

As shown in Fig. 6, the plane positions of the four bored cast-in-place piles in the two pile areas all changed with the progress of the construction process, that is, the positions shifted in the east–west and north–south directions. The black coordinate points in the figure indicate that the pile is offset in the north–south direction, and the positive offset indicates the northward offset, while the negative offset indicates the southward offset. Red coordinates indicate that the pile is skewed in the east–west direction, a positive offset indicates an eastward offset, and a negative offset indicates a westward offset. The straight line represents the allowable offset of the code. According to the Construction Code for Building Foundation Engineering, the allowable deviation range of the pile position of the injected pile is less than 100 + 0.01H, and H is the depth of the injected pile. According to the changes of the plane positions of pile J1 and J2 in Fig. 6a and b, it can be seen that the main deviation directions of the pile in the construction process are east and north, and their deviations meet the requirements of the specification. In Fig. 6c, the initial offset of H1 piles in both directions is lower than 35 mm, and the later offset increases rapidly to 100 mm, which is close to the maximum allowable offset value of the specification. In Fig. 6d, the offset direction of H2 pile is concentrated in the east direction, and its offset in the north direction is significantly lower than that in the east direction. In Fig. 6, the offset of cast-in-place piles increased much less in the early stage than in the later stage, which was consistent with the actual change of the project. The offset of the pile foundation above is within the standard range, it can be seen that the positioning accuracy of the pile foundation using the combined positioning technology is better.

Based on the data obtained by the biaxial inclination sensor, the verticality of all the intelligently constructed bored piles in the two pile areas was analyzed, the numerical simulation was carried out by Abaqus. The basis of meshing mainly considers the size of the pile and the calculation accuracy and efficiency. For piles of different sizes, corresponding meshing strategies are needed to fully reflect the geometric characteristics of the pile. Gridding needs to improve the computational efficiency as much as possible while ensuring the computational accuracy and minimizing the number of grids under the premise of meeting the accuracy requirements. The results were shown in Fig. 11.

The basis of meshing mainly considers the size of the pile and the calculation accuracy and efficiency during the numerical calculation. For piles of different sizes, corresponding meshing strategies are needed to fully reflect the geometric characteristics of the pile. Gridding needs to improve the computational efficiency as much as possible while ensuring the computational accuracy and minimizing the number of grids under the premise of meeting the accuracy requirements. Through reasonable mesh division, the stress state of the pile can be modeled more accurately to provide strong support for the actual project.

**Figure 7:**
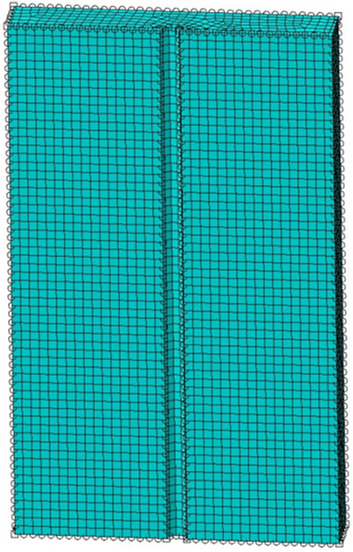
Column grid division diagram.

**Figure 8:**
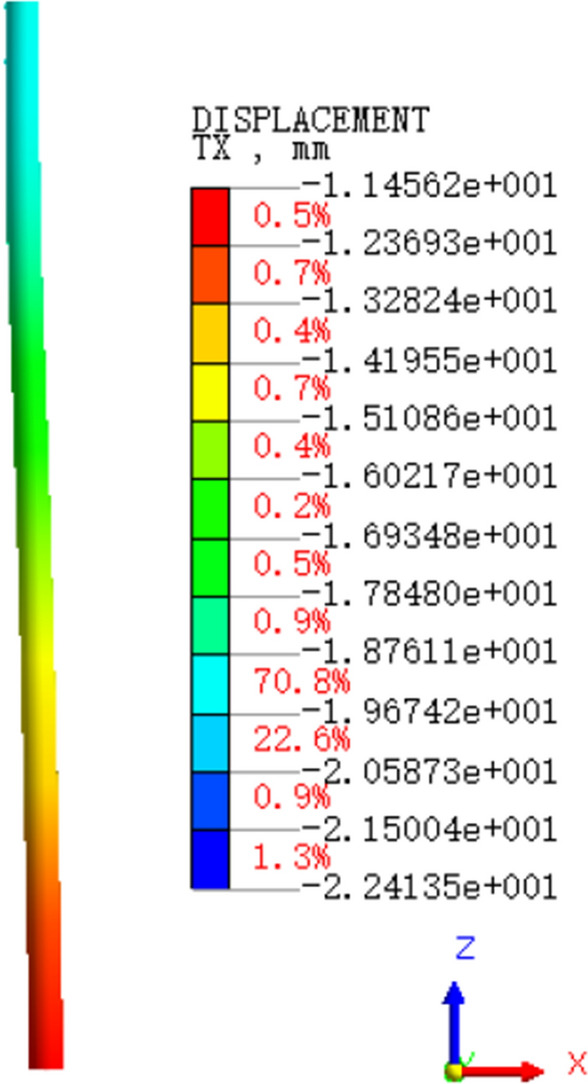
Cloud image of pile horizontal displacement.

**Figure 9:**
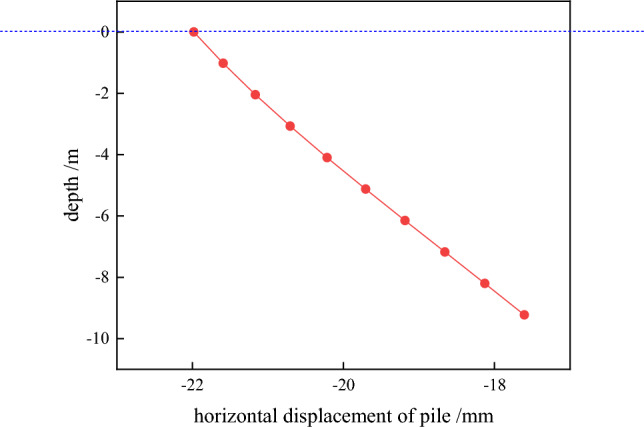
Horizontal displacement of pile.

**Figure 10:**
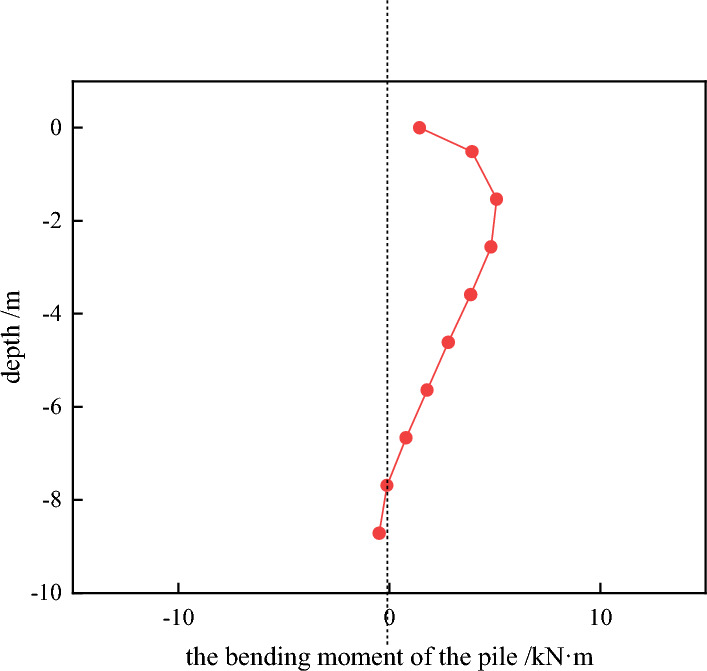
The bending moment of pile.

Through the numerical analysis to get the horizontal displacement of the pile cloud diagram. As shown in Fig. 7, we obtained the column grid division diagram and the cloud image of pile horizontal displacement(see Fig. 8). From Fig. 9 can be seen, with the increase of the depth of burial, the horizontal displacement of the pile shows a decreasing trend, and the two will be close to a linear relationship. Pile bending moment is also one of the factors to consider the stability of the structure. Through numerical calculation to get the rule of the pile bending moment with the depth of burial (shown in Fig. 10), when the depth of burial is less than 7.69 m the bending moment is positive, and with the increase of the depth of burial, the pile bending moment shows the trend of increasing and then decreasing. However, when the depth of burial is greater than 7.69 m, the pile bending moment is negative. Controlling the horizontal displacement and bending moment of the pile has a good guiding significance for the stability of the pile.

**Figure 11:**
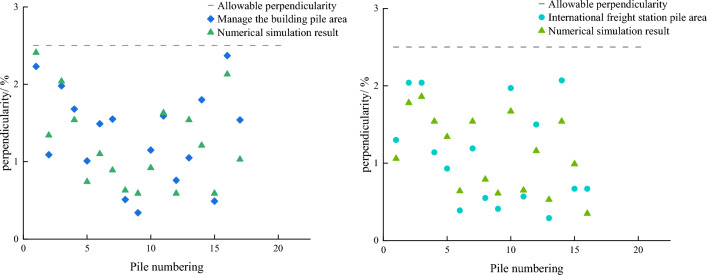
Vertical distribution diagram of pile foundation.

Pile perpendicularity is a parameter that describes the degree of deviation between the pile and the vertical direction. Specifically, it indicates the angle or deviation between the actual axis of the pile and the theoretical vertical axis. In pile foundation construction, the control of pile perpendicularity, which directly affects the bearing capacity and stability of the pile, is crucial. According to the "Construction Code for Building Foundation Engineering", it is known that the verticality of bored pile should be less than 1% in general, and the verticality of bored pile should be less than 2.5% when the drill pipe, guard barrel and guide barrel center are located in a straight line. As shown in Fig. 11, the verticality distribution of pile foundations in the two regions is discrete, in which the pile foundations with verticality below 1% in the pile area of the international freight station account for 56.3%, and those with verticality below 1% in the pile area of the management building account for 23.5%. The verticality of the overall pile foundation in the pile area of the international freight station is better than that in the pile area of the management building. The perpendicularity of both pile foundations is within the standard range. The numerical simulation results are also within the standard range. In conclusion, the intelligent monitoring system based on combined positioning technology and biaxial tilt sensor is well applied in the positioning of pile holes and the precision control of pile verticality.

## Conclusion

The construction process of bored pile is complicated and the engineering amount is huge, and the quality control of key processes in the construction process has significant influence on the quality of pile foundation. The accuracy of pile foundation hole positioning and the control of pile verticality are the key monitoring objects for the construction of bored piles. The existing traditional monitoring methods have shortcomings to varying degrees, and the monitoring data are easily affected by human beings, which makes the traditional monitoring methods unable to apply to the current pile foundation projects. In this study, an intelligent monitoring system for the construction of bored pile was constructed based on the combined positioning technology and biaxial inclination sensor. BDS and UWB technologies were used to monitor the plane position coordinates of pile foundation during hole formation in real time, the offset direction and offset of pile foundation were transmitted through wireless pulse information, and the perpendicularity of pile during pile formation was calculated through the biaxial inclination sensors on both sides of pile machine. The monitoring data is transmitted in real time and displayed on the digital construction integrated management platform to complete the whole project monitoring and abnormal early warning of bored piles. Through the application of Changshui Airport reconstruction and expansion project, it is found that the intelligent monitoring system is well applied in the determination of the plane position of bored pile and the control of pile inclination. Under the auxiliary operation of the system, the pile hole positioning and pile injection errors are kept within the relevant specifications, the numerical simulation effect is good, and the monitoring and warning of the key objects in the construction process of bored pile is realized.

## Data Availability

The data and materials used to support the findings of this study are available from the corresponding author upon request.

## References

[1] Gotman AL (2020). Pile foundations as an efficiently developing direction of foundation engineering. Soil Mech. Found. Eng..

[2] Wang XF, Li SX, Li JL (2022). Effect of pile arrangement on lateral response of group-pile foundation for offshore wind turbines in sand. Appl. Ocean Res..

[3] Maria I, Raffaele D, Luca D (2021). Serviceability analysis of piled foundations supporting tall structures. Acta Geotech..

[4] Wei YB, Cao Z (2019). Research and application of over-filling monitoring technology for underwater cast-in-place piles. IOP Conf. Ser. Earth Environ. Sci..

[5] Kam N, Seidman J, Lim R (2018). Examining auger cast-in-place piles in difficult ground conditions. Geotech. Special Publ..

[6] Wu DY, Qu BB, Zhao JH (2021). Application of intelligent management and control in the whole process of bored pile construction. Port Waterway Eng..

[7] Sun DW, Xu XN, Xu JC (2014). Optimization and application of rotary drilling techniques based on intelligent detection technology. J. Munic. Technol..

[8] He N, Ma GZ, He B (2021). Experimental study of sheet pile wharf based on distributed optical fiber monitoring technology. Hydro-Sci. Eng..

[9] Gao L, Zhou L, Liu HL (2021). Field experimental study on the high-precision measurement of deformation of cast-in-place pile. Rock Soil Mech..

[10] Zhang XS, Zhang XF, Han YS (2021). Optimization design and application of calibration method for overfilling monitoring system of cast-in-place pile. Sci. Technol. Eng..

[11] Wang L, Shu YL, Wu DY (2022). Research on the application of intelligent management and control technology in the construction of bored pile. China Water Transp..

[12] Liang N (2023). Practice of bored pile construction technology in road and bridge engineering. Sichuan Cem..

[13] Zhang YL, Huang HF, Si SY (2022). Research and application of super-long spiral drilled press-grouting pile technology. Yellow River.

[14] Cheng ZQ, Jiang Z (2023). Construction technology of bored cast-in-place pile for highway bridge pile foundation. Commun. Sci. Technol. Heilongjiang.

[15] Zou S, Li GZ (2019). Realizing digital boundary pile management system with BeiDou satellite system. Bull. Surv. Map..

[16] Zhu HW, Cai DG, Chen F (2017). Development and application of automatic monitoring system for pile foundation construction process. Railway Eng..

[17] Chai B, Chen YZ, Zhou JM (2021). Application of human-machine collaboration algorithm for mine pile weight estimation based on beidou high-precision location service. Lecture notes in electrical engineering.

[18] Xu JH, Zhang YL, Han YQ (2023). UWB indoor location method based on moving node auxiliary positioning. J. Chi. Inertial Technol..

[19] Zhang YK, Chun YX, Fu YF (2022). UWB positioning analysis and algorithm research. Procedia Comput. Sci..

[20] Shahi A, Aryan A, West J (2014). Deterioration of UWB positioning during construction. Autom. Constr..

